# Stability analysis of seed yield and agronomic performance of black cumin (*Nigella Sativa* L.) genotypes in Amhara Region, Ethiopia

**DOI:** 10.1371/journal.pone.0355682

**Published:** 2026-08-18

**Authors:** Azeze Wubie Kassa, Tegegne Ashagrie Endalew, Asnakew Takle Negash, Hayat Yasin Mohamed, Mulatie Kindu, Fentaw Semaw, Adargachew Demelashe, Melese Wale Mengistu, Endalkachew Akililu Tsemelesan, Atikilt Abera Alemayehu

**Affiliations:** 1 Gondar Agricultural Research Center, Gondar, Ethiopia; 2 Sirinka Agricultural Research Center, Woldya, Ethiopia; 3 Adet Agricultural Research Center, Bahir Dar, Ethiopia; Universidad Tecnica de Ambato, ECUADOR

## Abstract

Black cumin is an annual spice crop valued for its nutritional, medicinal and economical importance. Although the Amhara Region of Ethiopia possesses suitable agroecological conditions for black cumin production, seed yield remains below the national average, largely due to the limited availability of improved varieties. This study was conducted to evaluate the agronomic performance, genotype x environment interaction (GEI) and seed yield stability of promising black cumin genotypes. Multi-environment trials were carried out at three locations during the 2021 and 2022 main cropping using a randomized complete block design with three replications. Eighteen advanced genotypes and two standard check varieties were evaluated. Combined analysis of variance revealed significant differences among genotypes, environments, and genotype x environment interactions for all measured traits. Genotypes Moyale-230777 (G12), Achefer-9068 (G2), and Gofazuria-90517 (G7) exhibited the highest mean seed yields of 647.6 kg ha^-1^, 647.1 kg ha-1, and 623.9 kg ha-1, respectively. However, Achefer-9068 had lower oleoresin content. Moyale-230777(G12) and Gofazuria-90517 (G7) showed yield advantages of 17.8% and 26.8%, respectively, over the standard check varieties Dershaye and Aden. The genotype with environment interaction in the seed yield data of six environments (location and year combinations) was analyzed using AMMI and GGE biplot models. The result showed that environment had the greatest effect (53.0%) followed by GEI (32.2%) and genotype (14.9%). Based on AMMI, AMMI stability value (ASV), yield stability index, oleoresin content, and GGE biplot analysis G12 and G7 ranked the 1^st^ and 2^nd^ the genotypes for their yield performance, quality, identifying top performing genotypes with wide adaptability across the testing environments. Consequently, these genotypes were selected for on-farm verification, and accession number Moyale-230777 (G 12) has been released for commercial production in 2024 under the name “Tena”. Therefore, variety Tena is recommended for demonstration, scaling-up and commercial cultivation in the spice growing of Amhara Region and other similar agroecological zones.

## Introduction

Black cumin (*Nigella sativa* L.), is an important annual spice crop belonging to the family Apiaceae (Umbelliferae). It is widely cultivated in countries such as Iran, China, and Turkey and is believed to have originated in Egypt, the Eastern Mediterranean region, Southern Europe, and West Asia [1–3]. The plant is characterized by an erect and highly branched growth habit, is self-pollinated and has diploid chromosome number of 2n = 12 [3]. The flowers have five to ten petals, are fragile, and are often pale blue and white in color. The fruit is large, inflated capsule made up of three to seven joined follicles, each of which contains a significant number of seeds that are used as spices [4,5]. Black cumin is valued for its nutritional, medicinal, and economic importance. According to previous studies [1,6] Ethiopian black cumin accessions contain high levels of thymol, a monocyclic phenolic chemical that contributes significantly to the crop’s medicinal value. The seeds contain more than 100 identified chemical constituents, including essential oils and bioactive compounds with pharmaceutical and industrial applications [7]. In Ethiopia, black cumin is primarily cultivated for food flavoring, cosmetic and medicinal oil extraction, and export markets [8]. After ginger, it is the second-most significant cash crop sold to foreign markets [2]. Ethiopia possesses diverse agroecological zones that facilitate the production of a wide range of crops, spices, and vegetables [9,10]. Ethiopia is one of the top producers and consumers of spices, ranking first in Africa and seventh globally [10]. Many spice varieties, notably black cumin, white cumin, pepper, paprika, turmeric, fenugreek, garlic, coriander, ginger, cardamom, and basil, have been grown for consumption and commercial purposes [10].

Black cumin is mainly grown as a rain-fed crop in Ethiopia, mostly as residual moisture in the mid-to-highlands ranging from 1500−2500 m above sea level. The crop is widely cultivated in the Amhara, Oromia and the Southern Ethiopia regions, often under residual soil moisture conditions following the main rainy season. It is frequently interplant with cereals [11] and hot peppers (technical). Its ecological needs are comparable to those of chickpeas, fenugreek, and lentils, which typically grow with residual moisture conditions [12]. Black cumin has a small seed, and it needs well-prepared, fine, and well-drained soil for uniform and quick emergence. According to M. Getnet et al. [13], Ethiopia’s annual black cumin seed production is nearly 20 thousand metric tons. The productivity varies significantly depending on factors such as location, climate, soil type and agricultural practice, ranging from 640 kg ha ⁻ ¹ to 790 kg ha ⁻ ¹. Habtewold et al. [14], reported as seed yield of 790 kg ha⁻^1^, whereas Zigyalew [15] reported an average seed yield of 640 kg ha⁻^1^. The national average of black cumin productivity is well below the global average seed yield, although some countries like India achieve higher yield (2200 kg ha^-1^) as reported by [14]. There is no comprehensive or reliable information on the global average seed yield of black cumin, making it difficult to obtain accurate estimates from available sources. Despite its economic and medicinal importance, black cumin productivity in Ethiopia remains below its potential. Several factors contribute to the low productivity of black cumin in Ethiopia, including the limited availability of improved varieties, inadequate crop management practices, disease and insect pest pressure, poor plant population management, inadequate post-harvest handling, and weak market-oriented production systems, as reported by Ermias Assefa [16]. In the Amhara region, among the low yields and productivity, there is a shortage of improved varieties and spice crop research in the region has been neglected.

Crop performance is influence by genotype, environment, and the interaction between these factors. Genotype x Environment Interaction (GEI) plays a crucial role in determining the adaptability and stability of crop varieties across different growing conditions. Several statistical models have been developed and applied for studying genotype by environment effect and stability of genotype. Among these, Additive Main Effect and Multiplicative Interaction (AMMI) and GGE biplot models are widely used when the main effect and interaction both are important to increase accuracy for multi-environment trial data breeding program. AMMI model is used for the analysis and interpretation of both additive and multiplicative component of two data structure and powerful tools to analysis of GEI pattern by graphically [17–19]. This model integrated analysis of variance and principal component analysis (PCA) into united approach [19]. Similarly, GGE biplot analysis evaluates genotype performance by simultaneously considering genotype effects and genotype-by-environment interactions, providing a graphical approach for identifying superior and stable genotypes across environments [20]. In Ethiopia Black cumin genotypes were evaluated under multi-location trial in order to agronomic performance and stable genotypes, most of research work on nutritional and medicinal properties [21]. Information regarding genotype stability, adaptation patterns, and quality traits under the agroecological conditions of the Amhara Region remains limited. Therefore, the present study was conducted to evaluate the agronomic performance, yield stability, genotype × environment interaction, and oleoresin content of advanced black cumin genotypes across multiple environments. Therefore, the objective of the present study was to identify agronomic performance, stable seed yield, and good-quality black cumin genotypes and recommend the most promising ones for verification and variety release.

## Materials and methods

### Description of the study area

**Fig 1:**
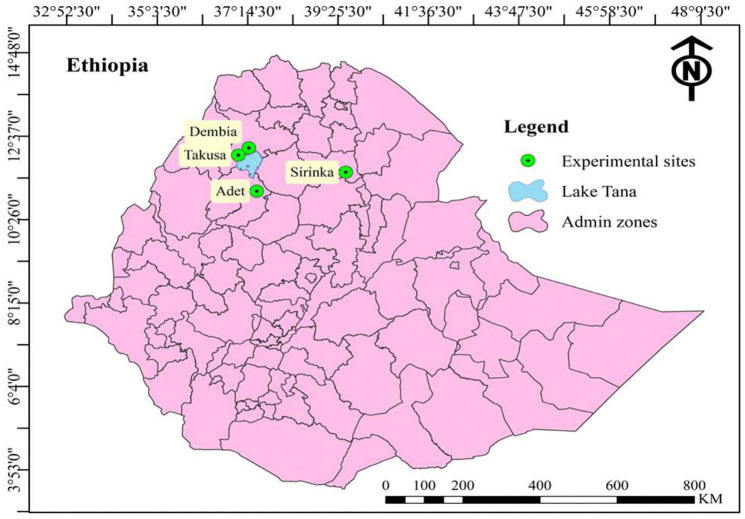
Map of experimental sites in Ethiopia. (Source: Generated by the author’s using DIVA-GIS shape files https://diva-gis.org/data.html).

**Fig 2:**
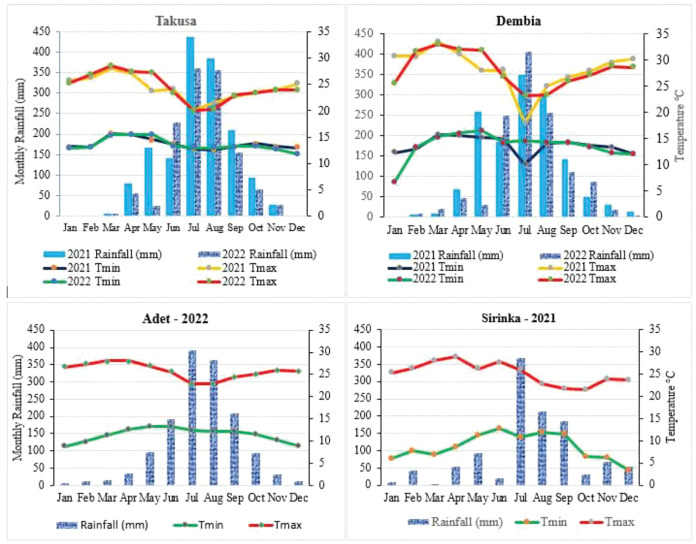
Monthly Rainfall and Temperature (Tmin and Tmax) of the experimental sites (Source Ethiopian metrological institute).

The experiment was conducted under rain-fed conditions during the 2021 and 2022 main cropping seasons at four representative black cumin-growing locations in Amhara region. Trials were established at Takusa and Dembia during both 2021 and 2022, while Sirinka and Adet were included in 2021 and 2022, respectively. The geographical locations and agroecology of the experimental sites were presented in Fig 1-2.

### Treatments and Experimental Setup

The seventy-two black cumin genotypes used in this study were obtained from the Ethiopian Biodiversity Institute (EBI) and consist mainly of landrace accessions collected from different agroecological zones of Ethiopia and evaluated in a nursery observation trial at the Takusa Research Station of Gondar Agricultural Research Center in 2019. The accessions consisted primarily of landrace collections in Ethiopia altitude range from mid to highland and exhibited erect, branched growth habits. Based on thier agronomic performance and seed yield, the promising accessions were advanced to a preliminary variety trial conducted at two locations during the 2020 cropping season. Eighteen superior genotypes (9067, 9068, 90508, 90509, 90511, 90516, 90517, 207539, 207540, 215319, 230038, 230777, 240403, 240404, 242223, 242842, 242836 and 242843) were advanced to regional variety trial with standard check Aden and Dershaye, in multi-environment trails. Description of the locations and genotypes were presented in Table 1 and Fig 2. The experiment was laid out in a Randomized Complete Block Design (RCBD) with three replications at each testing environment. Prior to sowing the land was prepared to a fine seedbed. Planting was done via seed drilling at a density of 10 kg ha ⁻ ¹. Each plot contained six rows spaced 30 cm apart. After emergence, seedlings were thinned to maintain 10 cm spacing between plants within rows. The net harvestable plot area was 3 m^2^. Fertilizers were applied as follows: 65 kg ha^-1^ of NPS (19-38-7) at planting and 74 kg ha^-1^ of Urea applied in two equal splits (at full emergence and at flowering). Weeds were controlled manually as required throughout the growing season. When infestations of bollworm and cutworm insects’ occurrence, Lambda cyhalothrin insecticide was applied at a rate of 0.4 L ha^-1^.

**Table 1:** List of genotypes/accessions and environments used for the experiment.

Entry	Genotype	Entry	Genotype	Location	Altitude	Env’t. code	Year	Soil type
G1	Achefer-9067	G11	Nensebo-230038	Takusa	1840	ENV1	2021	Vertisol
G2	Achefer-9068	G12	Moyale-230777	Takusa	–	ENV3	2022	Vertisol
G3	Jarso-90508	G13	Yeki-240403	Dembia	1920	ENV2	2021	Vertisol
G4	Agarfa-90509	G14	Yeki-240404	Dembia	–	ENV4	2022	Vertisol
G5	NS-90511	G15	Medebayzana-242223	Sirinka	1850	ENV5	2021	Vertisol
G6	NS-90516	G16	Sherka-242842	Adet	2240	ENV6	2022	Vertisol
G7	Gofazuria-90517	G17	Sherka-242836					
G8	Dembia-207539	G18	Sherka-242843					
G9	Laygaint-207540	G19	Aden					
G10	Enese-215319	G20	Dershaye					

### Data collection

Black cumin was harvested at physiological maturity, characterized by browning capsules and prior to seed shattering. Pods were sun-dried, and the seed yield per plot weighed using a sensitive balance and recorded in grams. Data were collected on agronomic and yield parameters, such as days to 90% maturity (MD), plant height (PH, cm), number of primary branches (BR), number of pods per plant, number of seeds per pod, seed yield (g plot^-1^), and finally seed yield converted to kg ha^-1^. The data for plant height, number of primary branches, and number of pods per plant were taken from the average of five randomly selected plants plot^-1^. The number of seeds per pod was counted manually.

Data for the quality parameter, the oleoresin content (percentage), and 300-gram seed for each genotype was measured. The Soxhlet extraction method was used [22] to quantify oleoresin content. Accordingly, the black cumin seed was ground using an electronic grinder (Panasonic, Japan, Model MJ-W176P), and the powder was packed in a polyethylene bag to prevent contamination until laboratory analysis was performed. The oleoresin was extracted with organic solvents using n-hexane and a Soxhlet apparatus for 2 h at 40–45 °C. The extracts were filtered and concentrated under reduced pressure using a rotary evaporator (model 4001 Rota vapor) to obtain crude extracts. The oil yield (%) was calculated as follows:


$$\begin{array}{c} \text{Oleoresin content }(\%)\;=\;(\text{yield of extract obtained }(\text{g}))/\;(\text{weight of sample used }(\text{g}))\text{ x100} \end{array}$$

### Ethics statement

This study did not involve human participants, animals, or human biological materials. The research consisted of field evaluation of black cumin (Nigella sativa L.) genotypes under standard agronomic practices. Therefore, ethical approval and informed consent were not required.

### Data analysis

Individual location and combined analysis of variance (ANOVA) were conducted across environments (locations and years) using R statistical software 4.5.2 [23]. Prior to a combined analysis, Bartlett’s test was used to confirm the homogeneity of error variances across environments. Treatments means were separated using the Least Significant Difference (LSD) test at a 5% probability level. The combined ANOVA model of fixed effects lines and random effects locations, years, and interactions was:


$$\begin{array}{c} A\text{NOVA model}:\;Y_{ijk}\;=\;\mu \;+\;G_{i}\;+\;E_{j}\;+{GE}_{ij}\;+\;Bk(j)\;+\;e_{ijk} \end{array}$$

Where *Y*_*ĳk*_ are observed values, µ is the grand mean, *G*_*i*_ is the effect of the *i*-th genotype, *E*_*j*_ is the effect of the j^th^ environment, *GE*_*ĳ*_ is the interaction effect, B_j_ and Bk(j) are block effects, and *e*_*ĳk*_ are the residual errors.

### Stability analysis

Genotype × Environment Interaction (GEI) was further investigated using the Additive Main Effects and Multiplicative Interaction (AMMI) model and the Genotype plus Genotype x Environment Interaction (GEI) biplot approach. AMMI analysis was conducted using the agricolae package in R, while GGE biplot analysis was performed using the metan package to evaluate genotype performance, stability, and adaptability across environments. Through AMMI model, GGE was further partitioned into IPCA components and the AMMI model according to Zobel et.al. [24] was used.


$$\begin{array}{c} {\text{Y}}_{\text{ij}}=\mu +{\text{G}}_{\text{i}}+{\text{E}}_{\text{j}}+\sum \;\lambda_{\text{k}}\;{\text{a}}_{\text{ik}}\;{\text{Y}}_{\text{jk}}\;+\;{\text{e}}_{\text{ij}} \end{array}$$

Where;

**Yij** is the yield of the i^th^ genotype in the j^th^ environment,

**µ** is the grand mean,

**Gi** and **Ej** are the genotype and environment deviations from the grand mean respectively.

**ƛ**_**ĸ**_ is the Eigen value of the interaction principal component axis K; **αik** and **Yjk** are genotype and environment principal component scores for axis K and **eij** is the error term.

Furthermore, AMMI’s Stability Value (ASV) and Yield Stability Index (YSI) were calculated in order to rank genotypes in terms of stability using the formula calculated by Purchase et al. [25], genotypes with lower ASV and YSI values were considered more stable across environments.

Stability Value (ASV) was calculated as:


$$\begin{array}{c} \text{ASV}=\sqrt{{\left[\frac{SS\;IPCA\text{1}}{SS\;IPCA\;\text{2}}\times IPCA\text{1}\;Score\right]}^{\text{2}}+(IPCA\text{2}\;Score{)}^{\text{2}}} \end{array}$$

Where, ASV = AMMI stability value; SS = sum of square; IPCA1 and IPCA2 = the first and the second interaction principal component axes, respectively and


$$\begin{array}{c} \text{YS}{\text{I}}_{\text{i}}=\text{ RAS}{\text{V}}_{\text{i}}\;+\text{ R}{\text{Y}}_{\text{i}} \end{array}$$

Where, YSI = yield stability index, RASV = Rank AMMI stability value and RY = rank of mean yield genotypes

## Results and discussion

### Seed yield and seed yield related traits

Analysis of variance for across six environments (combination of locations and years) revealed that seed yield differed significantly (*P < 0.001* and *P < 0.01*) among genotypes across the tested environments, indicating differential genotype responses to environmental conditions (Table 2). This showed that genotype might not express the same seed yield performance at the specific tests location. The highest-yielding genotypes differed among environments, due to the presence of genotype x environment interaction. The top performers in ENV1 (Takusa 2021) were G8 (Dembia-207539), G6 (NS-90516), G10 (Enese-215319), and G12 (Moyale-230777), with no statistically significant difference between them. G10 (Enese-215319), G12 (Moyale-230777), and G13 (Yeki-240403) were the highest performers in ENV2 (Dembia 2021), demonstrating notable genotype differences. The highest performances in ENV3 (Takusa 2022) were G2 (Achefer-9068), G4 (Agarfa-90509), and G7 (Gofazuria-90517), with notable variations between them. ENV4 (Dembia 2022): In terms of seed output, G20 (Dershaye), G11 (Nensebo-230038), and G3 (Jarso-90508) were the best. ENV5 (Sirinka 2021): With highly significant genotype differences, the top performers were G2 (Achfer-9068), G1 (Achefer-9067), and G7 (Gofazuria-90517) and ENV6 (Adet 2022): With extremely significant genotype differences, G12 (Moyale-230777), G9 (Laygaint-207540), and G7 (Gofazuria-90517). Several genotypes exhibited relatively broad adaptation across environments, maintaining superior performance despite environmental variability shown comparatively wider adaptation despite the variance. Their yields were consistently higher than both the genotypes mean and, most likely, the check variety.

**Table 2:** The mean seed yield (kg ha^-1^) of 20 black cumin genotypes tested at six environments.

Genotype code	Genotype Name	ENV1	ENV2	ENV3	ENV4	ENV5	ENV6
G1	Achefer-9067	710.8	514.6	596.9	654.9	624.8	563.7
G2	Achefer-9068	685.0	513.6	759.5	678.3	645.4	600.6
G3	Jarso-90508	668.4	477.5	409.8	697.9	363.9	645.2
G4	Agarfa-90509	675.9	531.6	665.1	602.2	338.7	402.3
G5	NS-90511	636.4	560.2	469.5	369.3	293.8	652.1
G6	NS-90516	775.7	543.5	569.8	650.1	346.4	648.4
G7	Gofazuria-90517	825.3	488.8	683.1	534.0	550.0	662.3
G8	Dembia-207539	736.4	466.2	453.3	527.3	312.0	498.7
G9	Laygaint-207540	689.5	543.9	451.3	637.3	362.6	691.8
G10	Enese-215319	756.8	683.8	449.1	664.2	424.3	512.9
G11	Nensebo-230038	719.7	413.3	623.1	741.3	329.5	511.5
G12	Moyale-230777	746.8	634.7	595.5	613.7	539.0	756.0
G13	Yeki-240403	666.8	568.3	524.7	644.1	338.5	612.2
G14	Yeki-240404	731.5	449.7	482.4	617.0	394.1	453.8
G15	Medebay zana-242223	734.4	413.9	508.0	639.7	493.0	624.5
G16	Sherka-242842	576.2	532.2	637.6	450.3	318.3	424.6
G17	Sherka-242836	660.4	347.7	469.3	543.1	382.4	529.7
G18	Sherka-242843	704.3	486.1	610.2	584.6	518.6	556.5
G19	Aden	657.6	416.6	555.1	668.2	350.3	416.2
G20	Dershaye	650.1	419.7	481.8	773.9	423.2	547.6
	Mean	700.4	500.3	549.8	614.6	417.4	565.5
	CV (%)	12.8	16.6	19.4	15.2	21.0	15.3
	LSD (0.05)	148.0	137.6	175.9	154.0	165.6	143.3
	Sig 5%	NS	**	**	**	***	***

Where *, ** and *** indicates significance at 0.05, 0.01 and 0.001 probability levels respectively, CV = Coefficient of variation, ENV’T = Environment and LSD = Least Significance Deference, ENV1 = Takusa 2021, ENV2 = Dembia 2021, ENV3 = Takusa 2022, ENV4 = Dembia 2022, ENV5 = Sirinka 2021 and ENV6 = Adet 2022

The overall mean yield performance genotypes an individual location ENV1 was better environment followed by ENV4, ENV3, ENV6, ENV2 and ENV5 respectively. This ranks Takusa as the most favorable environment under the conditions of this study, followed by Dembia, Sirinka, and Adet. In general, because of pod borer infestation, the overall yield performance of genotypes was low as compared with the national average (640−790 kg ha^-1^) and high-producing countries in the world such as India (2200 kg ha^-1^) on average. The current investigation demonstrated a greater environmental component contribution to the seed yield difference, comparable findings were published by outers Fufa et al. [21], Asefa and Beriso [26], Shoa et al. [27], Fikre et al. [28], Ejigu et al. [29] and Amdie et al. [30] these reported the genotypes’ mean yield varied between locations.

Combined analysis of variance for seed yield of black cumin genotypes evaluated across locations and genotype by environment were significant Table 3. Seed yield of black cumin genotypes across environments significant. Genotypes Moyale-230777 (G12), Achefer-9068 (G2), and Gofazuria-90517 (G7) showed the highest mean seed yields of 647.6 kg ha^-1^, 647.6 kg ha^-1^and 623.9 kg ha^-1^, respectively. However, Achefer-9068 (G2) showed had poor oleoresin content (Table 4). From the results, two genotypes, G12 (Moyale-230777) and G7 (Gofazuria-90517), were found to be superior better oleoresin content and had 17.8% and 26.8% yield advantage than standard checks, Aden and Dershaye respectively. Previous studies by Fufa et al. [21], Ejigu et al. [29] and Fikre et al. [28] indicated that there were highly significant differences among the genotypes in seed yield and oleoresin content of black cumin in multi-location tests.

**Table 3:** The combined analysis of variance for seed yield of 20 black cumin genotypes across environments.

Sources of variation	Degree of freedom	Sum square	Mean square	P value	F value
GEN	19	830319.5	43701.0	5.0	***
ENV	5	2801263.2	560252.6	64.0	***
GEN: ENV	95	1931824.0	20335.0	2.3	***
ENV: REP	12	402275.4647	33522.95539	3.8	***
Residuals	228	1995550.929	8752.416354	–	–

Where, GEN=genotype, ENV=environment, and REP= replication

**Table 4:** Combined ANOVA and mean values of seed yield and related traits of 20 Black cumin genotypes tested at six environment.

Genotype Code	Genotype Name	DMA	PLH (cm)	BR	PPP	SPP	SYLD (kg)	Oleoresin content (%)
G1	Achefer-9067	127	47.8	6.0	19	75	610.9	22.1
G2	Achefer-9068	129	49.5	5.4	18	77	647.1	31.3
G3	Jarso-90508	128	50.3	5.1	17	76	543.8	41
G4	Agarfa-90509	129	50.3	5.3	17	69	536.0	32.7
G5	NS-90511	128	48.3	5.5	16	74	496.9	25.6
G6	NS-90516	129	50.1	5.8	17	76	589.0	32.0
G7	Gofazuria-90517	129	48.0	5.8	20	76	623.9	46.4
G8	Dembia-207539	129	50.3	5.7	19	74	499.0	39.1
G9	Laygaint-207540	129	49.5	5.6	18	80	562.7	27.4
G10	Enese-215319	129	49.3	6.1	19	75	581.9	19.6
G11	Nensebo-230038	129	49.0	6.1	17	76	556.4	40
G12	Moyale-230777	127	46.5	6.4	21	77	647.6	48.7
G13	Yeki-240403	128	50.3	5.2	18	74	559.1	40.7
G14	Yeki-240404	128	48.6	5.5	19	75	521.4	40.1
G15	Medebayzana-242	128	49.0	5.7	20	71	568.9	17.8
G16	Sherka-242842	127	48.0	4.9	17	73	489.9	37.3
G17	Sherka-242836	128	49.1	5.5	16	79	488.8	41.9
G18	Sherka-242843	128	50.7	5.2	19	74	576.7	43.4
G19	Aden	126	50.6	5.8	16	76	510.7	36
G20	Dershaye	128	48.5	5.2	19	71	549.4	47.8
	Mean	128	49.2	5.7	18	75	558.0	
	CV (%)	1.7	6.5	16.0	20.14	12	16.7	
	LSD (0.05)	1.4	2.12	0.6	2	6.0	61.4	
	GEN	**	**	***	***	*	***	
	ENV	***	***	***	***	***	***	
	GEN: ENV	**	NS	***	***	NS	***	

Where *, **and *** indicates significance at 0.05, 0.01 and 0.001 probability levels, respectively. DMA = Days to Maturity, PLH = Plant Height, BR = Branch, PPP = Pod per Plant, SPP = Seed per Pod, SYLD = Seed yield, CV = Coefficient of variation, GEN = Genotype, ENV = Environment and LSD = Least Significance Deference

Seed yield related traits of black cumin genotypes evaluated across locations were significant difference (*P < 0.001* and *P < 0.01*). Number of primary branches and number of pod per plant were significant (*P < 0.001*), days to 90% maturity and plant height significant (*P < 0.01*), while seed per pod were significant (*P < 0.05*). Days to 90% maturity, plant height, number of primary branch, capsule (pod) number plant^-1^ and number of seeds pod^-1^, was ranged from 126 to 129, 46.5–50.6, 4.9–6.4, 16–21 and 69–80 respectively (Table 4). The highest number of capsules (pods) plant^-1^ (21) was recorded in G12, followed by G15 and G7, and G8 (20), while the lowest value (16) was obtained from G17 and G5. The highest number of seeds per pod was recorded in G9 (80), followed by G17 (79), G12 and G2 (77), and the lowest value was recorded G4 (69). Consistent with these research, Ermias [16], Fufa et al. [21], Asefa and Beriso [26], Shoa et al. [27], Fikre et al. [28], Ejigu et al. [29], Seid and Gedamu [31] and Amdie et al. [30] found that black cumin genotypes significantly affect the number of capsules plant^-1^, the number of seeds capsule^-1^, and days to 90% maturity.

### Oleoresin content

Differences in oleoresin content among the black cumin genotypes were determined. The highest oleoresin content was recorded in G12 (Moyale-230777) 48.7% followed by G7 (Gofazuria-90517) 46.4%. The lowest oleoresin content (17.8%) was obtained for G15 (Medebay zana-242223) (Table 4). The present results showed that G12 (Moyale-230777) and G7 (Gofazuria-90517) were superior in their oleoresin content and had an advantage of 26% and 22%, respectively, over Aden. Overall, the oleoresin yield of the tested genotypes ranged from 17.8% to 48.7%. This study in agreement with Fikre et al. [28] who has reported oleoresin content ranging from 31.17 to 43.24%. The variance could be caused by genetic and environmental factors, such as soil type, temperature, and altitude according to [23,25,30,33]

### Genotype x environment interaction (GEI)

A significant GEI indicated that different settings caused changes in genotype ranking orders. The highly significant GEI justified the use of stability analyses to identify broadly adapted and stable genotypes to know which component of the interaction is contribute more to the variation. The AMMI stability analysis of variance for seed yield revealed that the differences for the main effects of genotypes (G) and environments (E) and genotype x environment interactions (GEI) were all highly significant (*P < 0.01*). The environment had the greatest effect on the environmental sum of squares (53.0%) compared to genotype (14.9%) and GEI (32.2%) effects (Table 5). Environmental effects and GEI accounted for a larger proportion of the total variation than genotype effects alone.

**Table 5:** AMMI analysis of variance for seed yield (kg ha^-1^) of 20 genotypes tested across locations and years.

Source of Variation	Degree of freedom	Sum square	Mean square	Sum of squares Explained (%)		F value	P value
				Total	GEI		
Environments	5	2801263.2	560252.6	52.97	53.0	45.7	***
Genotypes	19	830319.5	43701.0	67.83	14.9	3.4	***
GEI	95	1931824.0	20335.0	100.00	32.2	2.3	**
IPCA1	23	649235	28227.6	33.6	33.6	3.2	***
IPCA2	21	551887.7	26280.4	62.2	28.6	3.0	***
IPCA3	19	404835.4	21307.1	83.1	21	2.4	***
IPCA4	17	183747.5	10808.7	92.6	9.5	1.2	0.246
IPC5	15	142118.4	9474.6	100	7.4	1.1	0.376
Residuals	214	3187706.7	13282.1	-	-	NA	NA

The observed GEI may therefore be partly explained by these seasonal climatic variations. Genotypes that performed consistently across years could be considered more stable and better adapted to fluctuating environmental conditions. These findings highlight the importance of considering climatic factors, particularly rainfall and temperature, when evaluating genotype performance across multiple environments (Fig 1 and 2).

### AMMI stability analysis

The AMMI analysis of variance for Black cumin indicated that the first, second and third AMMI components were significant (*P < 0.05*).The IPCA1 AMMI analysis explained 33.6%, whereas the IPCA2 analysis explained 28.6%. The IPCA1 and IPCA2 had sum square greater than that of the genotypes and cumulatively contributed to 62.2% of the total GEI (Table 5). The highly significant environmental effect and its high variance components could be attributed to the large difference between the tests locations in altitude, daily temperature and difference in both amount and distribution of rainfall (Fig 1 and 2). The previous report on black cumin in high land area of Ethiopia and other country also indicated that environmental effects has the largest parts of the total variation Fufa et al. [21], Asefa and Beriso [26], Shoa et al. [27], Fikre et al. [28], Ejigu et al. [29] and Amdie et al. [30]. Other crops like White cumin and Common bean in Ethiopia also indicated that environment has great effect on the total variation [31–35].

The mean, AMMI stability value and yield stability index were described in (Table 6). AMMI stability value and yield stability index are quantify and rank genotypes based on the stability parameters. According to both methods, the genotypes with the lowest ASV and YSI values were considered the most stable, as reported by Purchase et al. [25]. Based on the AMMI stability value, Sherka-242836, Dembia-207539 and Enese-215319 were most stable genotypes, however those genotypes had low yield and poor oleoresin content. Based on the Yield Stability Index (YSI), Enese-215319, NS-90516, and Moyale-230777 were identified as the most stable genotypes across environments. Based on AMMI stability value, yield stability index, and quality (oleoresin content) genotype, Moyale-230777 (G12) was better than the other genotypes.

**Table 6:** Performance and stability of 20 Black cumin genotypes based on mean grain yield (kg ha^-1^), IPCA1, IPCA2 scores, AMMI stability value (ASV) and Yield Stability Index (YSI).

Genotype	Yield	Yield Rank	IPCA1	IPCA2	ASV	ASV Rank	YSI (%)
Achefer-9067	610.9	4	−4.609	−1.321	5.55	9	13
Achefer-9068	647.1	2	−8.444	−4.135	10.71	19	21
Jarso-90508	532.7	14	5.342	6.647	9.12	18	32
Agarfa-90509	524.9	15	−5.746	−2.132	7.05	11	26
NS-90511	496.9	18	9.582	−8.457	14.04	20	38
NS-90516	589.0	6	3.736	1.202	4.53	5	11
Gofazuria-90517	623.9	3	−1.675	−6.879	7.15	12	15
Dembia-207539	499.0	17	2.634	0.169	3.09	3	20
Laygaint-207540	562.7	8	7.486	2.484	9.10	17	25
Enese-215319	593.0	5	4.144	2.254	5.35	8	13
Nensebo-230038	556.4	11	−4.727	5.717	7.95	14	25
Moyale-230777	647.6	1	4.944	−4.098	7.09	10	11
Yeki-240403	559.1	9	4.016	1.328	4.88	6	15
Yeki-240404	510.3	16	−1.849	2.793	3.53	4	20
Medebayzana-242223	557.8	10	−0.119	2.187	2.19	2	12
Sherka-242842	489.9	19	−3.093	−8.048	8.82	15	34
Sherka-242836	488.8	20	−0.682	0.844	1.16	1	21
Sherka-242843	576.7	7	−3.364	−3.166	5.05	7	14
Aden	532.9	13	−5.517	3.972	7.58	13	26
Dershaye	538.3	12	−2.059	8.640	8.97	16	28

### GGE biplot analysis

#### Mean ranking and stability of genotypes across environment.

**Fig 3:**
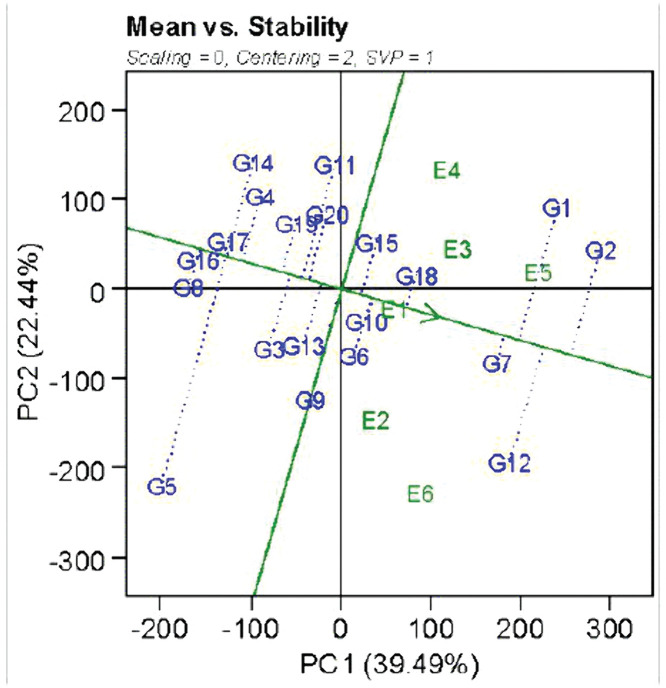
Mean seed yield performance and stability of genotypes based on the GEI data.

The analysis in earlier variety trials primarily focused on the main effects of genotypes (G), often considering GEI as random noise or confounding elements as noted by Yan and Tinker [36]. However, recent advancements have introduced several techniques aimed at analyzing GEI and evaluating genotype stability across diverse environments as highlighted by Shiferaw et al. [16,37], Fufa et al. [21], Asefa and Beriso [26], Shoa et al. [27], Fikre et al. [28], Ejigu et al. [29] and Amdie et al. [30]. One such approach is the GGE biplot method, which employs various biplot interpretation techniques to analyze both test environments and genotypic performance Yan et al. [18]. In this study, genotypes were evaluated using the “mean vs. stability” feature of the GGE biplot to assess their average performance and stability (Fig 3). The ENV (environment) and genotype codes were described (Table 1).The graph’s x-axis runs through the origin of the biplot and the marker representing the average environment, calculated based on the mean PC1 and PC2 scores across all test environments. The stability and average performance of the genotypes were measured by their projection onto the Y-axis and X-axis, respectively according to Weikai Yan [38]. Therefore, G2, G12, and G7 exhibited the highest average seed yields and were the most stable genotypes across the test environments.

### Which-won-where of genotypes

**Fig 4:**
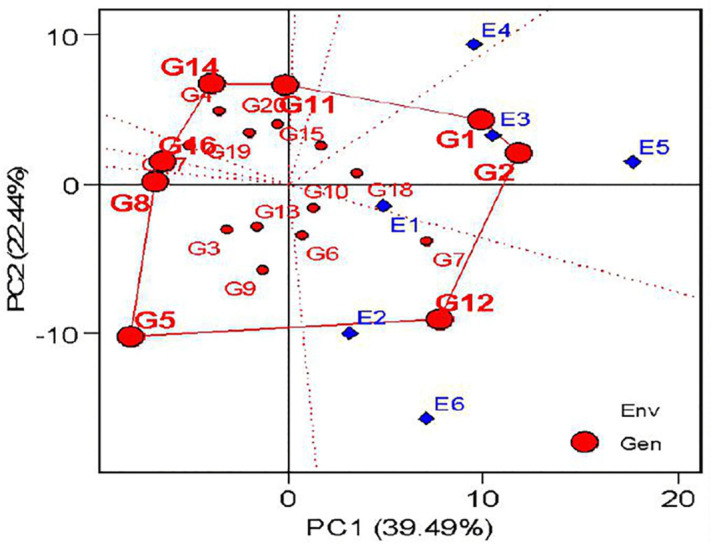
The which-won-where view of the GGE biplot of seed yield of black cumin genotypes based on the G × E data.

The “which-won-where” pattern of a genotype by environment data set is an important feature of GGE biplot. GGE model showed that the six environments used to the study grouped to three mega-environments. The biplot contains a polygon drawn on genotypes that are furthest from the biplot origin so that all other genotypes are contained within the polygon. These genotypes located on the vertices of the polygon performed either the best or the poorest in one or more environments according to Yan & Tinker [36]. The six environments fell in three sectors with different winning genotypes (Fig 4). Mega-environment 1 consists of three environments (E1, E2 and E6) that have good yield performance for genotypes G12, G7, G6 and G10, with the vertex genotype of this section being G12, a high-yielding genotype that performed well across most environments, indicating wider adaptability. Mega-environment 2 consists of two environments (E3 and E5) with good yield performance for genotypes G1, G2 and G18, with the vertex genotype of this section being G2. Mega-environment 3 consists of one environment (E4) with genotype G15 showing adaptation to this environment, with no vertex of genotype in this section. The two genotypes G12 and G2 were located on the vertex of the polygon and performed best in their respective environments. G12 was the winner in environments ENV 1, 2, and 6, while G2 was more adapted to testing environments ENV 4, 3, and 5. Environments within the same section have the same winning genotype, while environments in different section have different winning genotypes. Therefore, G7, G6 and G10 appeared to be near the origin of the biplot, performing moderately on average and these genotypes were less responsive to environments than the vertex genotypes. The check genotypes G19 (Aden) and G20 (Dershaye) and the other genotypes were vertex genotypes with no specific testing location adaptation (Fig 4). In this study, the portioning of G x E through GGE biplot analysis showed that PC1and PC2 accounted for 39.49% and 22.44% of the GGE sum of squares respectively, and explained 61.93 of the total variance.

#### Evaluation of genotypes relative to an ideal genotype.

**Fig 5:**
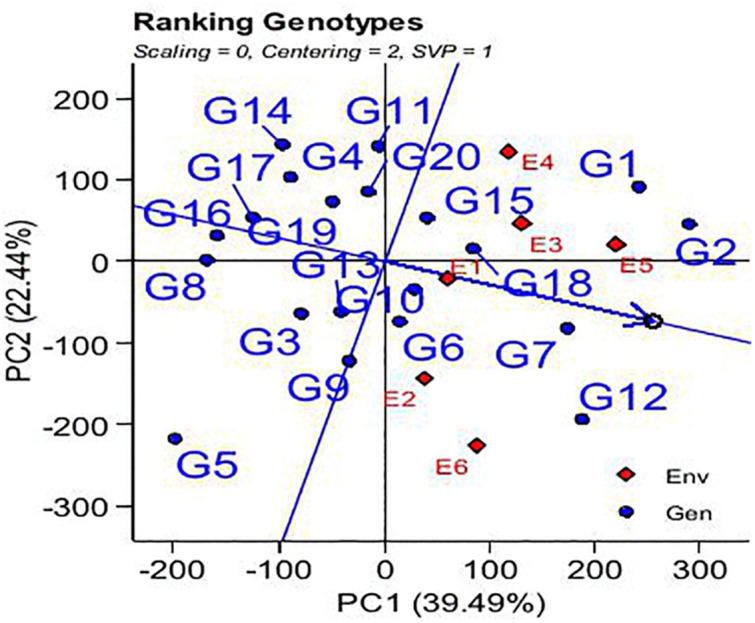
The ranking of the genotypes based on the ideal genotypes using GGE Biplot.

An ideal genotype (most stable and high mean yield) should be placed on the nearest to the center of concentric circles. The G7, G12 and G2 based on ranking of genotype analysis (Fig 5), genotypes G12 and G7 were positioned closest to the ideal genotype in the GGE biplot followed by G2 and G1 respectively, indicating superior yield performance and stability, while the other genotypes were far from the ideal. According to Yan W. [38], the genotypes near the center of the concentric circle are more approximate and ideal (i.e., high yield and stable). Similarly, the released genotype (G12) exhibited good yield performance and stable.

## Conclusion and recommendation

The multi-environment evaluation of 18 black cumin genotypes and two standard check varieties revealed significant genotype, environment, and GEI effects for seed yield and related traits. Compared to the two standard checks, Aden and Dershaye, and with other genotypes, genotype G12 and G7 were found to have the highest seed yield and oleoresin content AMMI stability analysis and GGE biplot evaluation identified genotypes with superior yield performance and stability across environments. Based on the integrated results of AMMI analysis, AMMI Stability Value (ASV), Yield Stability Index (YSI), oleoresin content, and GGE biplot analysis, genotypes G12 (Moyale-230777) and G7 (Gofazuria-90517) were identified as high-yielding, stable, and widely adapted genotypes. Consequently, these genotypes were advanced for national variety verification and evaluation. Accordingly, field verification trials were executed over locations on farmer’s field and research station and the candidate variety, G12 (Moyale-230777) was officially released under a variety name ‘Tena’ and is recommended for black cumin growers in potential midland areas of Amhara region and other similar agro-ecologies.

### Agronomic and morphological character of the released variety

- Variety name: Tena (Moyale-230777)
- Agronomic and morphological characteristics
- Adaptation area: Mid agro ecology
**•** Altitude(m.a.s.l): 1800–2200
- Rainfall (mm): 800–1200
- Seed rate(kg/ha): 10
- Spacing (cm): 30 between rows and 10 between plants
- Planting date: Mid-August to early September
- Fertilizer rate(kg/ha):
  - ◦ P2O5: 46
  - ◦ N: 60
- Days to maturity: 127
- Plant height(cm): 46.5
- Primary branches per plant: 6
- Number of pods per plant: 21
- Number of seed per pod: 77
- Growth habit: Erect &condensed branch
- Seed color: Deep black
- Flower color: White
- Oleoresin content (%): 48.7
- Crop pest reaction*: No observed disease; there were occurrence of cutworm and pod borer
- Yield (qt/ha)
  - ◦ Research field: 6-9
  - ◦ Farmers field: 6-9
- Year of released: 2024
- Breeder/maintainer: Gondar ARC/ARARI
